# Comparing the impact of future cropland expansion on global biodiversity and carbon storage across models and scenarios

**DOI:** 10.1098/rstb.2019.0189

**Published:** 2020-01-27

**Authors:** Amy Molotoks, Roslyn Henry, Elke Stehfest, Jonathan Doelman, Petr Havlik, Tamás Krisztin, Peter Alexander, Terence P. Dawson, Pete Smith

**Affiliations:** 1 Institute of Biological and Environmental Sciences, University of Aberdeen, 23 St Machar Drive, Aberdeen AB24 3UU, UK; 2 Stockholm Environment Institute York, Department of Environment and Geography, University of York, York YO10 5NG, UK; 3 School of Geosciences, University of Edinburgh, Edinburgh, UK; 4 Global Academy of Agriculture and Food Security, The Royal (Dick) School of Veterinary Studies, University of Edinburgh, Edinburgh, UK; 5 PBL Netherlands Environmental Assessment Agency, Bezuidenhoutseweg 30, 2594 AV The Hague, The Netherlands; 6 IIASA, Schlossplatz 1, A-2361 Laxenburg, Austria; 7 Department of Geography, King's College London, The Strand, London WC2R 2LS, UK

**Keywords:** land-use change, biodiversity, carbon storage, integrated models

## Abstract

Land-use change is a direct driver of biodiversity and carbon storage loss. Projections of future land use often include notable expansion of cropland areas in response to changes in climate and food demand, although there are large uncertainties in results between models and scenarios. This study examines these uncertainties by comparing three different socio-economic scenarios (SSP1–3) across three models (IMAGE, GLOBIOM and PLUMv2). It assesses the impacts on biodiversity metrics and direct carbon loss from biomass and soil as a direct consequence of cropland expansion. Results show substantial variation between models and scenarios, with little overlap across all nine projections. Although SSP1 projects the least impact, there are still significant impacts projected. IMAGE and GLOBIOM project the greatest impact across carbon storage and biodiversity metrics due to both extent and location of cropland expansion. Furthermore, for all the biodiversity and carbon metrics used, there is a greater proportion of variance explained by the model used. This demonstrates the importance of improving the accuracy of land-based models. Incorporating effects of land-use change in biodiversity impact assessments would also help better prioritize future protection of biodiverse and carbon-rich areas.

This article is part of the theme issue ‘Climate change and ecosystems: threats, opportunities and solutions’.

## 1. Introduction

Land-use change is a key direct driver of biodiversity loss [1,2] and is one of the main drivers of species extinctions [3]. It is also expected to be exacerbated by climate change, which can also impact indirectly on biodiversity in a number of ways [4]. For example, there is a negative global impact on crop production, which is projected to be high in the coming decades. For each degree-Celsius increase in global mean temperature, a 3.1–7.4% reduction in global yields of major crops is estimated [5]. This means cropland area will likely need to expand to meet the increasing demand for food [6,7], particularly in countries with growing food needs and limited access to technology for allowing sustainable intensification [8].

Cropland expansion is known to have severe adverse effects on natural biodiversity [9–11], through loss and fragmentation of habitats [12]. Conversely, land-use and land-cover change (LULCC) also impacts climate change and has accounted for an estimated 12.5% of anthropogenic carbon emissions from 1990 to 2010 [13]. Clearing natural ecosystems for crop production also releases carbon dioxide into the atmosphere as stored carbon is released from biomass and soil [14]. Human and natural responses to climate change are interconnected, with the majority of future model simulations of global cropland expansion exceeding the 15% planetary boundary in order to meet future food-supply targets [15]. Therefore, research on food production systems and ecosystem impacts should be prioritized [16].

Future land-use change has been explored through the application of modelling based upon the narratives for the shared socio-economic pathways (SSPs, [17,18]). Model results indicate a range of potential future land-use outcomes and have typically focused on consequences for greenhouse gas emissions, food provisions and prices. However, there has been less focus on potential consequences for biodiversity [19]. Furthermore, in a recent review of biodiversity scenarios, Titeux *et al*. [20] highlighted that biodiversity scenario analysis typically neglects consequences related to land-use change, but rather focuses on the direct impacts of climate change. Thus, exploration of biodiversity impacts of future land-use change scenarios, which are partially driven by climate change, warrants further research.

There are, however, large uncertainties associated with model-based projections of future global land-use change [21]. Existing studies have highlighted that both the total global quantity [22] and regional specific land-use changes [23] vary greatly according to the model. Similarly, other aspects such as climate change responses and bioenergy impacts [24,25] vary between models. While model inter-comparisons have considered differences in land-use change and associated climate impacts between models, no previous comparison has examined variation in biodiversity and carbon storage impacts. On a global scale, studies have shown a high correlation between species richness and carbon storage, with a strong association between carbon stocks and mammal, bird and amphibian distributions [26]. Although plot-level studies observe weaker correlations, a strong association has also been observed at a national level, with a high proportion of threatened species relying on carbon-rich habitats in tropical regions [27]. Cropland expansion threatens both carbon storage and biodiversity, with consequences for ecosystem functioning [7,28,29]. This global study therefore aims to compare the impact of cropland expansion projections on biodiversity and carbon storage across three different models and three different SSPs. This process allows the quantification of variability in biodiversity and carbon outcomes associated with model and scenario, which is important for more holistic assessments of the impact of land-use change. Differentiating the effect of extent and location can also be used to determine the relative importance of improving the accuracy of land-based models or socio-economic scenarios, for the purposes of prioritizing areas for biodiversity conservation and carbon storage in the future.

## 2. Material and methods

### (a) Land-use models

Model outputs from the modelling teams GLOBIOM [30], IMAGE [31] and PLUMv2 [32] were collected, each looking at the time period 2010–2050. GLOBIOM is a global recursively dynamic partial equilibrium model that integrates the agricultural, bioenergy and forestry sectors [30], with its main drivers being population, GDP, input prices, bioenergy demand, taxes and yields [33]. It requires geographical information and land profitability of crop production for its land allocation [30], basing its cropland expansion on a land rent approach [21]. In comparison, the land component of IMAGE [31] uses a computable general equilibrium model, MAGNET [34], to calculate agricultural demand, trade and production. There are six key drivers for IMAGE: demography, economic growth, policy and governance, technological development, culture and lifestyle, and natural resource availability, with a regression-based suitability assessment allocating land-use change [31]. PLUMv2 is a global land-use and food-system model that combines spatially explicit, biophysically derived yield responses with socio-economic scenario data to project future demand, land-use and management inputs [32]. For each country and time step, the agricultural land use and level of imports or exports are determined through a least-cost optimization that meets the demand for food and bioenergy commodities in each country. GLOBIOM uses the crop model EPIC [33] while IMAGE uses the dynamic global vegetation model LPJmL [35] to determine cropland yields, both producing a spatially explicit output at 0.5 × 0.5° gridded resolution. Similarly, PLUMv2 [32] uses a dynamic global vegetation model, LPJ-GUESS, to provide crop yield responses on a 0.5 × 0.5° grid [36].

### (b) Scenarios

The models described can be used to simulate the effects of different SSPs [32,37–39], which are defined as ‘reference pathways describing plausible alternative trends in the evolution of society and ecosystems over a century timescale’ [40]. SSP1 represents low challenges for mitigation and adaptation to climate change, SSP2 is moderate and SSP3 is high. SSP1 is the ‘greenest’ with sustainable development proceeding at a high pace, lessening global inequalities. There is a rapid technological change towards low carbon energy sources and high productivity of land, while SSP3 has a slow technological change, a rapidly growing population with unmitigated emissions. Investments in human capital are also low, with high inequality, reduced trade flows and large numbers of people being left vulnerable to climate change with low adaptive capacity. SSP2 is an intermediate case between SSP1 and SSP3 and represents a future where development trends are neither extreme, but follow a middle-of-the-road pathway consistent with typical patterns observed over the past century [41].

### (c) Biodiversity metrics

#### (i) Alliance for Zero Extinction sites

An Alliance for Zero Extinction (AZE) site is identified by three criteria: it must contain at least one individual species that has been evaluated as Endangered or Critically Endangered under the International Union for Conservation of Nature (IUCN) criteria; it is the sole area where this species occurs, containing over 95% of the known resident population; and it has a definable boundary [42]. These species often have little official protection, making them extremely vulnerable to external threats such as habitat destruction [43]. Currently, 587 sites for 920 species of mammals, birds, amphibians, reptiles, conifers and reef-building corals have been identified, with 81% of AZE sites being found within a biodiversity hotspot. These sites are therefore an important indicator of biological significance and the impact of future cropland expansion could threaten them further. So, the AZE dataset was used in a spatial overlay, as in Molotoks *et al*. [44], to examine infringement of cropland expansion on AZE sites. The sum of AZE sites per region was then calculated per model and per scenario to estimate the total number of sites impacted.

#### (ii) Conservation International hotspots

Cropland expansion projections within Conservation International (CI) hotspots were also explored, the criteria for these sites accounting for vascular plant species richness. CI hotspots identify regions of importance for biodiversity, and to qualify, a region must be threatened—i.e. contain at most 30% of its original natural vegetation—yet contain at least 1500 different species of endemic vascular plants. The 35 CI hotspots cover 2.3% of the land surface but support 50% of the world's endemic plant species and 43% of vertebrate endemic species [45,46]. CI hotspot shapefile data were converted to 0.5° raster maps. Any 0.5° cell containing CI hotspot polygon data is classified as a CI hotspot irrespective of hotspot size. The CI map is therefore binary and cells are classified as either a CI hotspot or not.

#### (iii) Vertebrate species-rich regions

As another biodiversity metric, maps of vertebrate species richness, small-range vertebrate species richness and threatened species richness were considered [3,47]. The resolution of the vertebrate species richness maps was decreased from 0.1 to 0.5° resolution to match the resolution of the three models involved in our analysis; the mean species richness was calculated for each grid cell. For all taxa, the distribution of species richness across grid cells is right-skewed: most cells contain a few species, while there are a few cells with a large number of species. For each taxon, therefore, the mean species richness values of grid cells were converted into percentile values and ‘species-rich regions' assumed to be cells in the 90th percentile of grid cells.

Cropland expansion projected by PLUMv2, IMAGE and GLOBIOM in vertebrate species-rich regions was explored across the three SSP scenarios. Furthermore, for each model and SSP combination, regions of threat—regions with high biodiversity (either CI hotspot or vertebrate species-rich region) under pressure from cropland expansion—were identified. An overall threat index for all species per grid cell was then calculated. This is the percentage of cropland expansion projected by 2050 from the models multiplied by the summed richness index of amphibians, birds and mammals. For the threat index, it was assumed that each species is equally important regardless of the taxon. Calculating the threat index allowed comparisons of the location of threatened areas between the models and SSPs.

### (d) Carbon storage

#### (i) Biomass

To examine storage loss in vegetation, cropland expansion projections for each model and scenario were overlaid with a combined dataset of carbon storage in 14 forest types [44]. Vegetation carbon stocks presented by Ruesch & Gibbs [48] for land covers represented in the Global Land Cover 2000 map [49] were used to calculate carbon loss at 1 km resolution in tonnes per hectare. This represents the total biomass carbon stored in both above- and below-ground vegetation. Where cropland expansion projections overlapped with forests, it is assumed the carbon stored is lost as a result of vegetation being cleared. Building on the methodology used in Molotoks *et al*. [44], the mean value of carbon present in tonnes per hectare, and the area and the percentage of cropland expansion for each individual grid cell were used to calculate an estimated total carbon loss.

#### (ii) Soil

Soil carbon stocks represented in the Harmonized World Soil Database [50] were also examined. Thirty arc second resolution grids for each land use represented in the Global Land Cover 2009 map were used [49], employing the total organic soil carbon stock density to a depth of 1 m reported by Hiederer & Köchy [51]. The mean value of carbon present for each grid cell, majority land cover, and figures from a global meta-analysis of land-use change impacts on soil organic carbon (SOC) [52] were used to calculate estimates of soil carbon loss. For example, there is an estimated 42 and 59% loss of SOC when forest and grassland, respectively, are converted to cropland [52].

### (e) Statistical analysis

A similar approach to the statistical analysis to that by Prestele *et al*. [23] and Alexander *et al*. [22] was taken, identifying the sources of variance in the results for each of the different biodiversity metrics considered, by fitting multiple linear regressions with model and SSP as variables. Interaction terms were not considered, and the variance associated with such interactions is incorporated within the residuals. An analysis of variance was then performed on the regression models to extract the type II sum of squares values for each variable to partition the relative importance of model and scenario. The statistical analysis here is not used to draw inferential conclusions with regard to whether the models or SSP scenarios have statistically significant effects on cropland expansion and, consequently, biodiverse regions. Rather, the variance of the results is partitioned to indicate the level of variance that can be associated with model choice or SSP scenario.

## 3. Results

To summarize, across all metrics, SSP1 typically has the lowest impacts on biodiversity and carbon storage. PLUMv2, in general, shows the least impact on carbon storage, while IMAGE has the highest impact across biodiversity metrics. The highest impact on carbon storage is also seen in IMAGE, but there is variation between carbon loss from biomass and from soil. For all metrics used, both for biodiversity and carbon storage, the majority of variance is explained by the model used (table 1).

**Table 1.** The proportion of variance explained by the model and SSP for each of the biodiversity metrics considered. The *R*^2^ value for the linear model for each metric is given. *p*-values are not used as linear models were not used to identify whether model or SSP has a statistically significant effect on the biodiversity metrics examined.

	proportion of variance explained by		
metric	model	SSP	*R*^2^
AZE sites	63.5	21.4	0.849
carbon loss from biomass	69.7	25.0	0.947
carbon loss from soil	62.1	27.2	0.893
amphibian spp.-rich hotspots	63.5	23.0	0.864
bird spp.-rich hotspots	75.3	19.7	0.949
mammal spp.-rich hotspots	68.3	22.1	0.904
CI hotspots	83.9	11.2	0.951

### (a) Biodiversity metrics

#### (i) AZE sites

**Figure 1.**
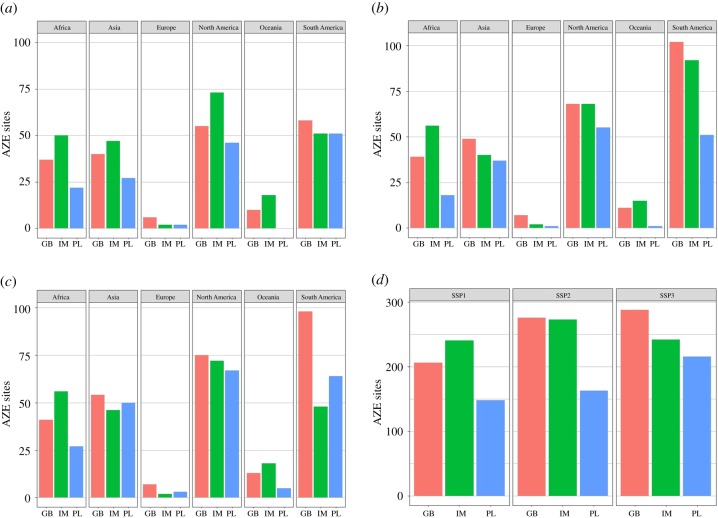
(*a–c*) The number of AZE sites impacted by cropland expansion between 2010 and 2050 for each region and model by socio-economic scenario (SSP1–3). (*d*) A comparison between models at a global level. GB, GLOBIOM; IM, IMAGE; PL, PLUMv2.

For all three models, cropland expansion infringing on AZE sites is lowest under SSP1 (figure 1*d*). In the SSP1 scenarios, IMAGE projections show the greatest impact on AZE sites globally, while in the SSP2 and SSP3 scenarios, GLOBIOM projections show the greatest impact (figure 1*d*). For example, in SSP2, 102 sites are projected to be impacted by cropland expansion in South America alone (figure 1*b*). PLUMv2 projections show the smallest impact across all scenarios at a global level and across most regions (figure 1). However, while there is variation in the number of AZE sites that cropland is projected to expand into across the SSPs, SSP accounted for only 21.4% of the variation in model results (table 1). A larger fraction of the variation (63.5%) in the AZE results is explained by the model (table 1).

Europe is almost consistently the region with the fewest sites impacted across all models and SSPs, while the Americas are the most highly impacted. South America has the highest numbers of AZE sites impacted by cropland expansion across all SSPs (figure 1*a–c*). There is, however, variation within the models. For example, IMAGE projections show higher numbers of AZE sites impacted in North America than South America for SSP1 and 3 (figure 1*a,c*). Similarly, PLUMv2 projects a slightly higher number of AZE sites impacted in North America than South America in SSP2 (figure 1*b*). There is also variation across other regions between model projections. IMAGE consistently projects the highest numbers of AZE sites impacted in Africa and Oceania across all three scenarios, while GLOBIOM projections show higher impacts for Europe and South America (figure 1*a–c*).

#### (ii) Vertebrate species-rich regions and CI hotspots

**Figure 2.**
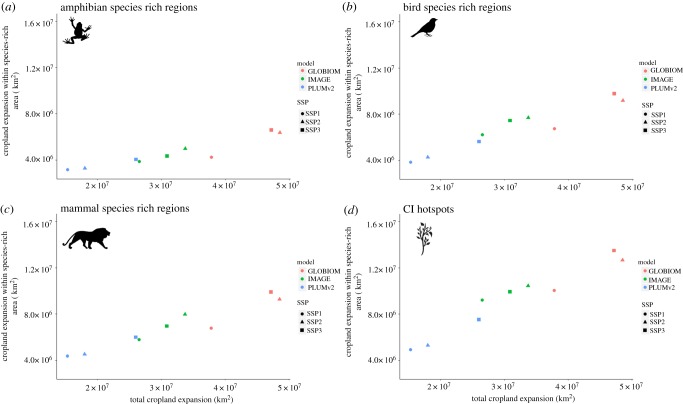
Projected cropland change between 2010 and 2050 in (*a*) amphibian, (*b*) bird, (*c*) mammal and (*d*) CI hotspots across the different SSP scenarios and models. Species-rich regions are composed of cells with a richness index greater than or equal to 0.9.

**Figure 3.**
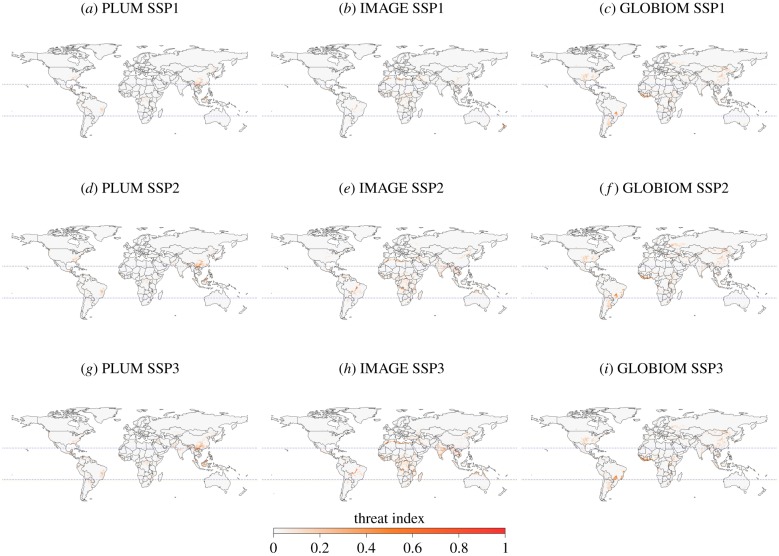
Spatial distribution of regions of threat: regions with high biodiversity under pressure from cropland expansion. Calculated in each 0.5° grid cell as the fraction of a grid cell converted to cropland between 2010 and 2050 multiplied by the summed richness index of birds, mammals and amphibians. The different SSPs are displayed in different rows and the different models are displayed in different columns. Blue dotted lines delineate the tropics.

As with AZE sites, the smallest areas of cropland expansion in vertebrate species-rich and CI hotspots regions are found in the SSP1 scenarios (figure 2). SSP3 has the largest impacts, projecting the greatest area (electronic supplementary material, figures S1–S4) with a high threat index in all three models (figure 3). Yet the majority of variation is explained by the model (table 1).

Figure 3 shows this variation between the models. South-east Asia is the most heavily affected in PLUMv2 projections, while West Africa and the Cerrado region in Brazil show the most cropland expansion in GLOBIOM projections (figure 3). GLOBIOM also projects the greatest levels of total cropland expansion in all species-rich regions under SSP2 and SSP3 (figure 2). For IMAGE projections, a wide range of areas in the tropics are shown to be affected, including Southeast Asia, Central Africa and the fringes of the Amazon rainforest in South America (figure 3).

### (b) Carbon storage

**Figure 4.**
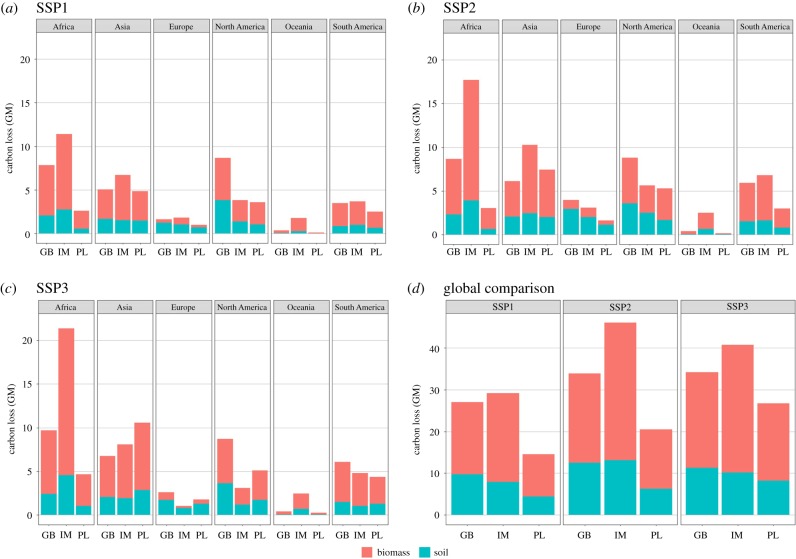
(*a–c*) Carbon loss in gigatonnes (Gt) from soil and biomass as a result of cropland expansion between 2010 and 2050 for each region and model by socio-economic scenario (SSP1–3). (*d*) A comparison between models at a global level. GB, GLOBIOM; IM, IMAGE; PL, PLUMv2.

For all three models, SSP1 has the lowest estimated carbon losses, for both the total estimates and individual estimates from biomass and soil, with the lowest estimates consistently shown in PLUMv2 projections (figure 4*d*). Across all scenarios, IMAGE projections show the highest total losses of carbon, with the greatest total estimate from SSP2 at over 46 gigatonnes of carbon (GtC) lost from soil and biomass combined (figure 4*d*). However, GLOBIOM generally has larger projected losses for soil carbon (figure 4*d*), with higher carbon loss from temperate regions, including North America (figure 4*a–c*).

As with biodiversity metrics, the model used also explains the greatest proportion of variance for carbon loss (table 1). Africa and Oceania consistently have the largest impacts from IMAGE projections, whereas Europe and North America have the highest losses from GLOBIOM, and PLUMv2 shows higher losses in Asia (figure 4*a–c*).

## 4. Discussion

SSPs are intended to have different environmental implications and therefore modelled differences between the SSPs are not unsurprising. While global land-use models differ by design, they all aim to model the same global system, capturing the same dynamics, and therefore ideally generate similar results under single scenarios. While all three models demonstrate some commonality in overall results, the models still vary considerably in their estimates of cropland expansion within SSPs. Our results are therefore in agreement with previous studies investigating uncertainties in land-use projections. For example, Alexander *et al*. [22] and Prestele *et al*. [23] both found large differences in land-cover projections between models, with the highest variability occurring in future cropland areas. Our study is the first to our knowledge, however, to consider the implications of similarities and differences in land-cover projections arising under different models for biodiversity and carbon. Highlighting uncertainties between modelling approaches in terms of biodiversity and carbon storage impacts is important for conservation goals and climate change mitigation. When informed by model outcomes, conservation or mitigation measures could be misled when uncertainty is not considered. Conversely, identifying similarities between models across different metrics will help to identify key regions for prioritization to ensure conservation and mitigation targets are met.

### (a) Biodiversity perspectives

The biodiversity results demonstrate similar broad patterns across models. For example, SSP1 consistently has the lowest levels of cropland expansion in AZE sites, vertebrate species-rich regions and CI hotspots across all models (figures 1 and 2). Our results therefore agree with the findings from Chaudhary & Mooers [19], who used land-use model projections from the land-use harmonization dataset (LUH2) and found SSP1 resulted in the lowest land-use change-driven global biodiversity loss. SSP1 is characterized by slow population growth, global sustainability and low vulnerability to climate change [53]. The world's growing population, coupled with increased affluence, is a major driver of food demand [54], so slow population growth will greatly influence the amount of cropland expansion. There is also strong land-use change regulation in SSP1 to avoid environmental trade-offs and large assumed improvements in agricultural productivity [17], which would limit cropland expansion and subsequent encroachment into biodiverse regions [55]. By contrast, across most models, the greatest levels of cropland expansion in biodiversity metrics examined are projected under SSP3, with the exception of IMAGE (figure 2). SSP3 is characterized by limited land-use regulation and continued deforestation; therefore, increased cropland expansion and subsequent changes in biodiverse regions are expected [17]. Given the agreement of the models for SSP1, it is important for conservation that policy decisions strive for a global future characterized by land-use change regulation and ‘green’ choices, protecting biodiverse regions from cropland expansion.

There are certain areas that the models agree will experience cropland expansion (figure 3, electronic supplementary material, figures S1–S4) within the species-rich regions and AZE sites. This agreement highlights them as areas of particular conservation concern. For example, under SSP3, areas of Central Africa that contain high numbers of mammal species would be at risk (electronic supplementary material, figure S3). In terms of AZE sites, the models all project the greatest number affected by cropland expansion will be those in the Americas (figure 1). Mexico (classified as North America in this study) is almost always the country with the highest numbers of AZE sites affected across all models, followed by Peru and Columbia (electronic supplementary material, appendix SA). Other studies have also identified Mexico as a country expected to experience large habitat declines for a number of species by 2050 due to food production and consumption increases [56]. High levels of species richness and a large number of AZE sites cluster in the tropics. High levels of cropland expansion are projected in these areas as well; therefore, the tropics and sub-tropics are where the threat index is found to be highest across the models (figure 3). The tropics are also likely to see the greatest benefit to biodiversity in terms of the most species preserved, if global warming is constrained from 1.5 to 2°C [4]. Hence, the indirect impacts of climate change via land-use change could affect similar areas to those experiencing direct impacts of climate change. In particular, previous studies have highlighted areas in Central and South America as global priorities for adaptation of both agriculture and biodiversity in the face of climate change [4,16,57]. The three models here all agree that cropland expansion is expected in the tropics, with notable impacts on AZE sites in the Americas. Thus, our results similarly suggest areas in Central and South America as conservation priorities, regardless of the SSP considered.

Despite similar general patterns across SSPs and some local spatial agreement in projected land change, there is considerable variability in the overall estimates of cropland expansion between models and, consequently, the effects on biodiverse regions and AZE sites (figures 1 and 2). Within SSPs, PLUMv2 consistently displays the lowest rates of cropland expansion, followed by IMAGE and GLOBIOM. Consequently, the impact of cropland expansion in AZE sites, CI hotspots and species-rich regions is lowest in projections produced by PLUMv2 and highest in projections produced by GLOBIOM. Furthermore, the larger cropland expansion with GLOBIOM results in larger areas of the temperate zones, such as North America, arising as regions of threat (figure 3) compared with PLUMv2 and IMAGE. The lower cropland expansion observed in PLUMv2 likely results from the inclusion of crop and location-specific fertilizer, irrigation intensification and the modelling of adaptation. GLOBIOM determines the amount of land that will be converted to agriculture through the use of a land supply curve [58]. It has a high estimate of cropland availability as it is based on estimates of land productivity, relying mainly on biophysical production constraints [30]; hence, it has the largest extent of cropland expansion estimates of the three models (figure 2).

### (b) Carbon perspectives

Similar to the biodiversity metrics, across models, SSP1 has the lowest estimated carbon losses. However, estimates of carbon losses differ considerably between models. PLUMv2 consistently projects the lowest levels of carbon loss while, despite greater global cropland expansion with GLOBIOM, IMAGE projects the highest estimates of total carbon loss across SSPs at a global level. This global-level effect is largely driven by the location of cropland expansion in IMAGE compared with the other models. IMAGE projects high rates of cropland expansion in Central Africa, where some of the largest intact areas of tropical forest cover are located [59,60]. Tropical vegetation currently stores approximately 340 billion tonnes of carbon and therefore higher rates of cropland expansion in Central Africa, as projected using IMAGE, result in higher levels of total carbon loss compared with the other models [14]. This finding corroborates previous work and demonstrates the importance of considering not only uncertainty surrounding the magnitude of global cropland expansion but also the spatial location [23]. Our results serve to highlight that the location of cropland expansion has implications for carbon storage and, hence, the prioritization of land conservation to mitigate carbon losses should consider the influence of models used to generate projections and the potential uncertainty involved.

Despite model differences, this study demonstrates that future cropland expansion has a significant negative impact on carbon storage. As much as 46 GtC is projected to be lost before 2050 (figure 4), which is 3.4 times greater than the current annual global anthropogenic greenhouse gas emissions [36], at a time when it is essential to minimize such emissions [61]. Although models vary in their global estimates of potentially available cropland [58], large areas of remaining potentially cultivatable land are currently beneath tropical forests [62]. Deforestation of the tropics for cropland expansion could lead to large-scale biodiversity and carbon losses. Although the feedback is not captured within all the models examined here, carbon loss contributes directly to climate change which, in turn, results in negative impacts on crop yield and increases the need for further cropland expansion. Consequently, future assessments of the impact of climate change on biodiversity and carbon storage should also consider the indirect effects of climate through land-use and land-management change [4].

### (c) Dealing with uncertainty in land-based modelling studies

Our aim is to demonstrate the similarities and differences between models and scenarios concerning the impact of cropland expansion on carbon storage and biodiversity metrics. Given the apparent agreement between models and different metrics, we have highlighted SSP1 as the most desirable scenario for both biodiversity and carbon storage, although this scenario still projects high future impacts on metrics examined. For example, between 14 and 30 GtC are projected to be lost in this scenario (figure 4*d*), 5–10 × 10^6^ km^2^ of CI hotspots converted to cropland (figure 2*d*) and up to 241 AZE sites impacted by this land-use change (figure 1*d*). This emphasizes the need for a redoubling of efforts in SSP1 to avoid severe environmental impacts of future cropland expansion. Furthermore, we have identified regions that could be considered as priorities for both biodiversity and carbon storage loss (e.g. the Americas). However, there remains considerable variability in the estimates of cropland expansion between models within individual SSPs (figure 2). Our results therefore demonstrate that intrinsic model characteristics can over- or underestimate cropland expansion irrespective of the scenario of interest. Model characteristics, parameterizations and institutional assumptions often lead to divergent land-use outcomes. Differences between the models here likely arise because of assumptions regarding cropland intensification, adaptation and estimates of cropland productivity. Furthermore, previous land-use model inter-comparisons have highlighted uncertainty arising from differences between initial land-use input data, bioenergy production assumptions and yield responses to climate change associated with the underlying crop models [21–24,63]. For example, Alexander *et al*. [22] found substantial differences in starting cropland areas across 17 different models. Models often allocate land-use change based on land use in adjacent grid cells in former time steps (e.g. cropland expansion at the edge of existing agricultural area). Therefore, starting conditions can have a large influence on the dynamics of cropland expansion in future time steps [22]. The models used here and in other comparison studies also have different underlying crop yield models. Hence crop yield responses to inputs such as fertilizer and climate change can differ and ultimately affect the area of cropland required to meet projected demand for crop production [63].

## 5. Conclusion

Here, we highlight firstly that even in the most environmentally sustainable pathway, there are significant impacts on biodiversity and carbon storage. Hence, the importance of going beyond measures taken in the SSP1 scenario is emphasized. Secondly, the existence of uncertainty in land-use change projections needs to be acknowledged when designing conservation or mitigation strategies. Models are frequently selected for biodiversity or carbon studies based on user familiarity and accessibility, but rarely are the results from more than a single model considered. Our intention is not to identify model results that are more plausible or the most accurate model. However, we show that it would be beneficial to include a range of models and scenarios when studying land-use effects on biodiversity and carbon such that model uncertainty can be explored and areas for prioritization identified. This is particularly important for prioritizing AZE sites as the vast majority are unprotected, yet host small, restricted populations [43] of endemic, rare and threatened species [64]. They are particularly vulnerable to external threats, as 95% of each individual species are found within the boundaries of their site [42]. Hence, increased accuracy of land-based modelling studies could help prioritize sites to protect, thereby reducing potential future species extinctions. Recent studies have urged caution when using a single model for estimates of land-use change for environmental assessments [23]; here, we would urge the same from a biodiversity and carbon storage perspective. Previous efforts to model scenario outcomes, representative concentration pathways (RCPs) or SSPs, on biodiversity may also benefit from reassessment within the context of other land-use models to generate uncertainty. Focusing conservation efforts and climate mitigation in regions where models agree there will be substantial impacts could be an effective approach to conservation. Furthermore, considering results across different types of metrics (e.g. species-rich regions, AZE sites and carbon stocks) could provide a comprehensive picture of biodiversity and carbon storage impacts, allowing a holistic and cost-effective approach to prioritization.

## Supplementary Material

Supplementary information and figures

## Data accessibility

This article has no additional data.
