# Supplementary material for: Comparing the impact of future cropland expansion on global biodiversity and carbon storage across models and scenarios
Source: Philos Trans R Soc Lond B Biol Sci. 2020 Jan 27;375(1794):20190189. doi: 10.1098/rstb.2019.0189 (PMC7017773; doi:10.1098/rstb.2019.0189)
Supplement: Supplementary information and figures [file rstb20190189supp1.docx]

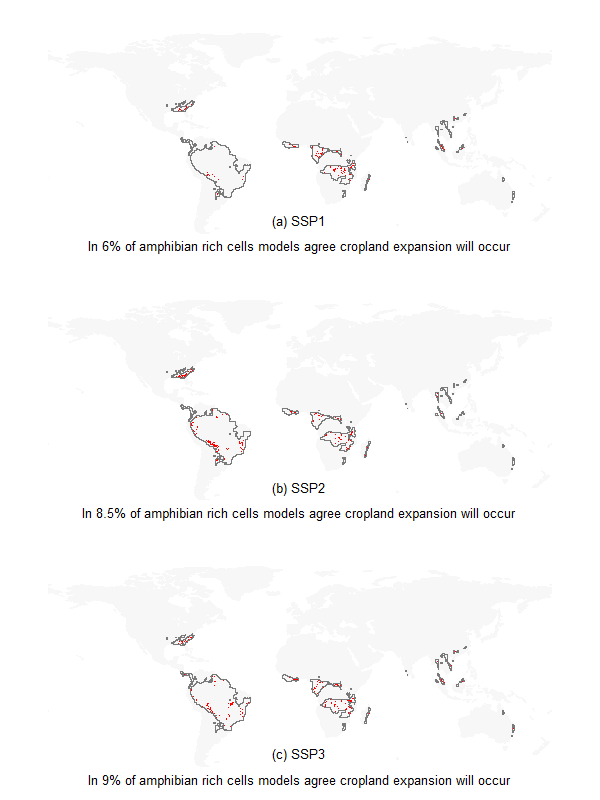


*Figure S1: Spatial distribution of agreement across models on where cropland will occur from 2010-2050 within amphibian species rich hotspots across SSP scenarios*


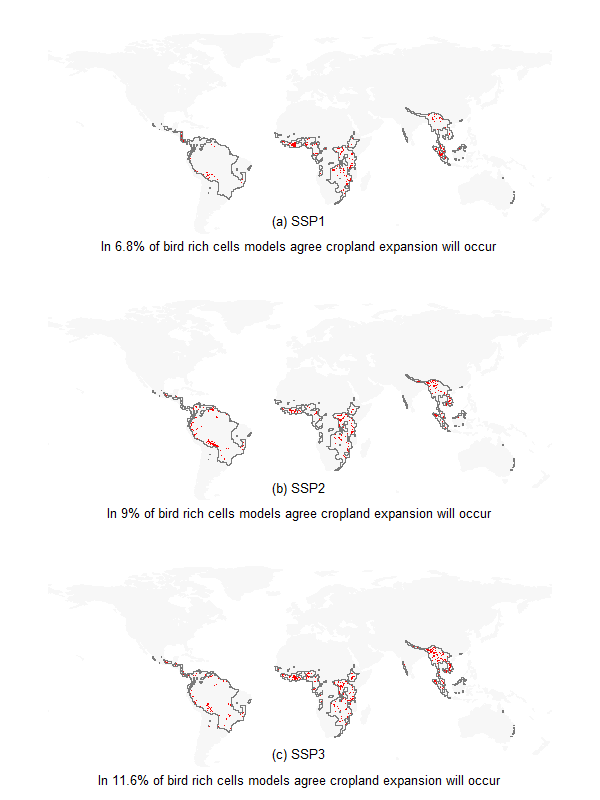


*Figure S2: Spatial distribution of agreement across models on where cropland will occur from 2010-2050 within bird species rich hotspots across SSP scenarios*


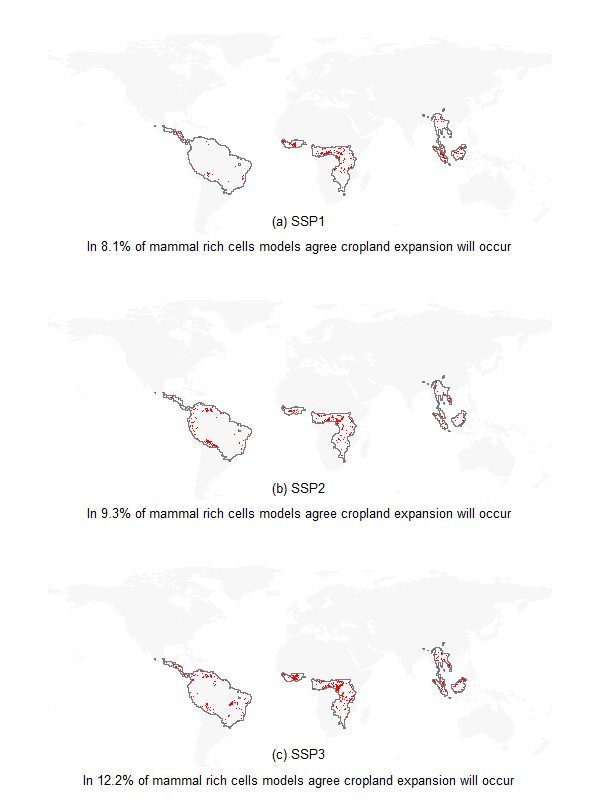


*Figure S3: Spatial distribution of agreement across models on where cropland will occur from 2010-2050 within mammal-species-rich hotspots across SSP scenarios*


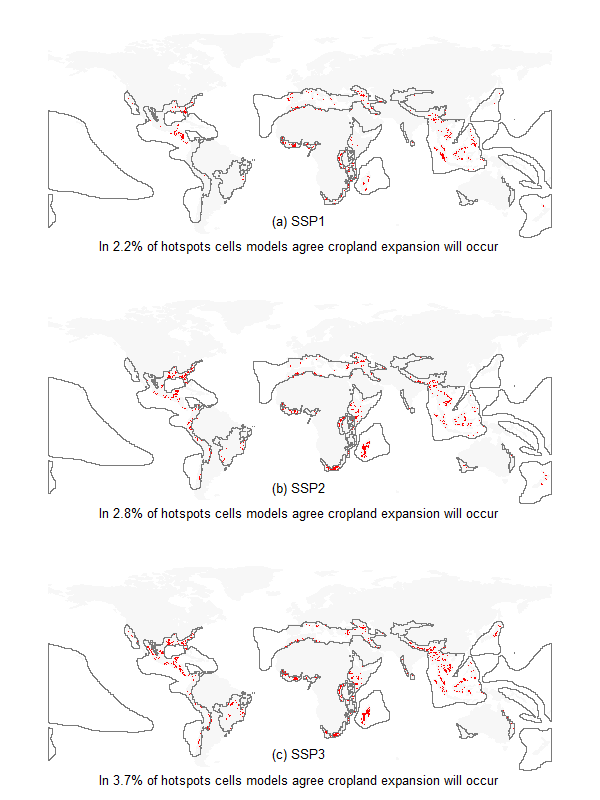


*Figure S4: Spatial distribution of agreement across models on where cropland will occur from 2010-2050 within CI biodiversity hotspots across SSP scenarios*

*Appendix A: Count of AZE sites per country impacted by cropland expansion from 2010-2050 across all models and SSP scenarios. Rows with more than one country indicate AZE sites overlapping multiple countries.*

|  | SSP1 | | | | | Total | SSP2 | | |  | ssp3 | | | Total | Grand total |
| --- | --- | --- | --- | --- | --- | --- | --- | --- | --- | --- | --- | --- | --- | --- | --- |
|  | GloBIOM | | IMAGE | PLUM | |  | GloBIOM | IMAGE | PLUM Total | | GloBIOM | IMAGE | PLUM |  |  |
| Africa | 37 | | 50 | | 22 57 | | 39 | 56 | 18 63 | | 41 | 56 | 27 | 62 | 66 |
| Angola | 2 | | 1 | | 2 | 2 | 2 | 2 | 2 | 2 | 2 | 2 | 2 | 2 | 2 |
| Cameroon | 3 | | 4 | | 1 | 6 | 3 | 9 |  | 9 | 6 | 9 | 1 | 9 | 9 |
| Congo, The Democratic Republic Of | 1 | | 3 | | 2 | 3 | 1 | 3 | 1 | 3 | 2 | 3 | 2 | 3 | 3 |
| Cote D'ivoire | 1 | | 1 | |  | 1 | 1 | 1 |  | 1 | 1 | 1 |  | 1 | 1 |
| Cote D'ivoire, Guinea, Liberia | 1 | | 1 | | 1 | 1 | 1 | 1 | 1 | 1 | 1 | 1 |  | 1 | 1 |
| Ethiopia | 4 | | 4 | |  | 4 | 5 | 5 |  | 6 | 4 | 4 |  | 5 | 6 |
| Etiopia |  | |  | |  |  | 1 |  |  | 1 |  |  |  |  | 1 |
| Gabon |  | |  | | 1 | 1 |  |  | 1 | 1 |  |  | 1 | 1 | 1 |
| Kenya | 3 | | 4 | | 2 | 4 | 3 | 3 | 2 | 4 | 3 | 3 | 2 | 4 | 4 |
| Liberia | 1 | | 1 | |  | 1 | 1 |  |  | 1 | 1 | 1 |  | 1 | 1 |
| Madagascar | 10 | | 17 | | 5 | 18 | 10 | 18 | 2 | 19 | 10 | 20 | 9 | 20 | 20 |
| Malawi | 1 | |  | |  | 1 | 1 | 1 |  | 1 | 1 |  |  | 1 | 1 |
| Mozambique | 1 |  | | | | 1 |  |  |  |  |  |  |  |  | 1 |
| Reunion |  | |  | |  |  |  | 1 |  | 1 |  |  |  |  | 1 |
| Réunion (to France) |  |  | | | |  |  | 1 |  | 1 |  | 1 |  | 1 | 1 |
| Rwanda | 1 | | 1 | |  | 1 | 1 | 1 |  | 1 | 1 | 1 |  | 1 | 1 |
| Somalia | 1 | |  | |  | 1 | 1 | 1 |  | 1 | 1 | 1 | 1 | 1 | 1 |
| South Africa | 1 | | 1 | | 1 | 1 | 1 | 1 | 1 | 1 | 1 | 1 | 1 | 1 | 1 |
| Tanzania, United Republic Of | 3 | | 7 | | 6 | 7 | 4 | 6 | 6 | 6 | 3 | 6 | 6 | 6 | 7 |
| Uganda | 2 | | 2 | |  | 2 | 2 | 2 | 1 | 2 | 2 | 2 | 1 | 2 | 2 |
| Zimbabwe | 2 | | 2 | | 1 | 2 | 2 |  | 1 | 2 | 2 |  | 1 | 2 | 2 |
| ASIA | 40 | | 47 | | 27 | 72 | 49 | 40 | 37 | 76 | 54 | 46 | 50 | 78 | 86 |
| Afghanistan |  |  | | | |  | 1 | 1 |  | 1 | 1 |  |  | 1 | 1 |
| China | 8 | | 13 | | 13 | 21 | 9 | 8 | 14 | 21 | 10 | 11 | 14 | 21 | 23 |
| India | 5 | | 7 | | 2 | 10 | 11 | 11 | 6 | 14 | 12 | 14 | 7 | 15 | 16 |
| Indonesia | 12 | | 14 | | 3 | 18 | 13 | 11 | 1 | 18 | 14 | 12 | 12 | 20 | 20 |
| Japan | 4 | | 1 | | 1 | 4 | 4 | 3 | 2 | 5 | 3 | 2 | 3 | 3 | 6 |
| Malaysia | 2 | | 2 | | 2 | 2 | 2 |  | 2 | 2 | 2 |  | 2 | 2 | 2 |
| Myanmar | 2 | |  | | 2 | 3 | 2 | 2 | 2 | 3 | 2 | 2 | 2 | 3 | 3 |
| Philippines | 1 | | 4 | | 1 | 4 | 1 | 1 |  | 2 | 3 |  | 1 | 3 | 5 |
| Sri Lanka | 6 | | 3 | |  | 6 | 6 | 2 | 6 | 6 | 6 | 3 | 6 | 6 | 6 |
| Vietnam |  | | 3 | | 3 | 4 |  | 1 | 4 | 4 | 1 | 2 | 3 | 4 | 4 |
| EUROPE AND THE MIDDLE EAST | 6 | | 2 | | 2 | 7 | 7 | 2 | 1 | 7 | 7 | 2 | 3 | 8 | 8 |
| Armenia | 1 | | 1 | | 1 | 1 | 1 | 1 |  | 1 | 1 | 1 | 1 | 1 | 1 |
| Austria |  | |  | | 1 | 1 | 1 |  | 1 | 1 | 1 |  | 1 | 1 | 1 |
| Italy | 1 | |  | |  | 1 | 1 |  |  | 1 | 1 |  |  | 1 | 1 |
| Portugal | 1 | |  | |  | 1 | 1 |  |  | 1 | 1 |  |  | 1 | 1 |
| Spain | 1 | |  | |  | 1 | 1 |  |  | 1 | 1 |  | 1 | 1 | 1 |
| Turkey | 2 | | 1 | |  | 2 | 2 | 1 |  | 2 | 2 |  |  | 2 | 2 |
| Ukraine |  | |  | |  |  |  |  |  |  |  | 1 |  | 1 | 1 |
| NORTH AMERICA | 55 | | 73 | | 46 | 103 | 68 | 68 | 55 | 113 | 75 | 72 | 67 | 113 | 128 |
| America | 1 | | 2 | | 1 | 2 | 1 | 2 | 1 | 2 | 1 | 2 | 1 | 2 | 3 |
| Canada | 2 | | 1 | |  | 2 | 1 |  | 1 | 1 | 1 | 1 | 1 | 1 | 2 |
| Costa Rica | 3 | | 2 | | 2 | 3 | 3 | 1 | 2 | 3 | 3 | 2 | 2 | 3 | 3 |
| Costa Rica, Panama | 1 | | 1 | | 1 | 1 | 1 |  | 1 | 1 | 1 | 1 | 1 | 1 | 1 |
| Cuba | 2 | | 5 | | 3 | 6 | 3 | 5 | 3 | 10 | 3 | 12 | 6 | 14 | 14 |
| Dominican Republic | 2 | 2 | | | | 2 |  |  | 2 | 2 |  | 1 | 2 | 2 | 2 |
| El Salvador, Honduras | 1 | |  | |  | 1 | 1 |  |  | 1 | 1 |  |  | 1 | 1 |
| Guadeloupe | 1 |  | | | | 1 |  |  |  |  |  |  |  |  | 1 |
| Guatemala | 8 | | 6 | | 6 | 8 | 8 | 5 | 6 | 8 | 8 | 6 | 6 | 8 | 8 |
| Haiti | 5 | |  | | 2 | 5 | 5 |  | 4 | 6 | 6 |  | 4 | 6 | 6 |
| Honduras | 10 | | 6 | | 6 | 12 | 11 | 4 | 4 | 12 | 11 | 9 | 9 | 12 | 13 |
| Jamaica | 2 | | 2 | |  | 3 | 5 |  |  | 5 | 5 |  |  | 5 | 5 |
| Mexico | 14 | | 44 | | 21 | 49 | 23 | 45 | 28 | 51 | 28 | 36 | 33 | 50 | 57 |
| Panama | 1 | |  | | 1 | 1 | 1 | 1 | 1 | 1 | 1 | 1 | 1 | 1 | 1 |
| Puerto Rico | 3 | |  | |  | 3 | 3 |  |  | 3 | 3 |  |  | 3 | 3 |
| St Lucia |  | | 1 | |  | 1 |  |  |  |  |  |  |  |  | 1 |
| Trinidad and Tobago |  | | | | |  |  | 1 |  | 1 | 1 |  |  | 1 | 1 |
| United States Of America | 2 | |  | | 1 | 3 | 2 | 4 | 2 | 6 | 2 | 1 | 1 | 3 | 6 |
| PACIFIC | 10 | | 18 | |  | 23 | 11 | 15 | 1 | 23 | 13 | 18 | 5 | 25 | 31 |
| Australia | 4 | | 9 | |  | 12 | 5 | 7 | 1 | 12 | 6 | 8 | 4 | 13 | 14 |
| Fiji |  | | 2 | |  | 2 |  | 1 |  | 1 |  |  |  |  | 2 |
| New Caledonia | 1 |  | | | | 1 |  | 3 |  | 3 |  | 3 | 1 | 3 | 3 |
| New Zealand | 2 | | 2 | |  | 2 | 2 | 2 |  | 2 | 2 | 3 |  | 3 | 3 |
| Papua New Guinea | 4 | | 2 | |  | 4 | 4 | 1 |  | 4 | 5 | 4 |  | 6 | 6 |
| Samoa |  | |  | |  |  |  | 1 |  | 1 |  |  |  |  | 1 |
| Solomon Islands | 1 |  | | | | 1 |  |  |  |  |  |  |  |  | 1 |
| Vanuatu |  | | 1 | |  | 1 |  |  |  |  |  |  |  |  | 1 |
| SOUTH AMERICA | 58 | | 51 | | 51 | 103 | 102 | 92 | 51 | 138 | 98 | 48 | 64 | 124 | 153 |
| Argentina | 4 | | 2 | | 2 | 5 | 5 | 5 | 4 | 8 | 5 | 4 | 5 | 9 | 9 |
| Bolivia | 8 | | 4 | | 2 | 8 | 9 | 6 | 2 | 9 | 9 | 4 | 2 | 9 | 9 |
| Brazil | 6 | | 17 | | 8 | 22 | 12 | 8 | 6 | 14 | 12 | 3 | 8 | 15 | 24 |
| Chile | 1 | | 1 | |  | 1 | 1 | 3 |  | 3 | 3 | 1 | 1 | 3 | 3 |
| Colombia | 9 | | 10 | | 7 | 17 | 24 | 21 | 13 | 33 | 24 | 6 | 16 | 29 | 35 |
| Colombia, Venezuela | 1 | 1 | | | | 1 |  | 2 |  | 2 |  | 2 | 1 | 2 | 2 |
| Ecuador | 6 | | 7 | | 8 | 11 | 12 | 16 | 8 | 23 | 10 | 3 | 5 | 13 | 23 |
| Paraguay |  | |  | | 1 | 1 |  |  | 1 | 1 |  |  | 1 | 1 | 1 |
| Peru | 19 | | 6 | | 18 | 29 | 30 | 22 | 14 | 34 | 27 | 18 | 20 | 32 | 35 |
| Venezuela | 5 | | 3 | | 4 | 8 | 9 | 9 | 3 | 11 | 8 | 7 | 5 | 11 | 12 |
| Grand Total | 206 | | 241 | | 148 | 365 | 276 | 273 | 163 | 420 | 288 | 242 | 216 | 410 | 472 |
